# Cognitive function, mood and sleep changes in response to a Tai Chi/Qigong intervention among older breast cancer survivors: an exploratory analysis

**DOI:** 10.3389/fcogn.2024.1402873

**Published:** 2024-08-14

**Authors:** Dara L. James, Molly Maxfield, SeungYong Han, Nanako A. Hawley, Megan E. Petrov, Dorothy D. Sears, David E. Vance, Linda K. Larkey

**Affiliations:** 1 Center for Health Promotion and Disease Prevention, Edson College of Nursing and Health Innovation, Arizona State University, Tempe, AZ, United States,; 2 Edson College of Nursing and Health Innovation, Arizona State University, Tempe, AZ, United States,; 3 Department of Research and Evaluation, Kaiser Permanente Southern California, Pasadena, CA, United States,; 4 Department of Psychology, University of South Alabama, Mobile, AL, United States,; 5 College of Health Solutions, Arizona State University, Tempe, AZ, United States,; 6 School of Nursing, University of Alabama Birmingham, Birmingham, AL, United States

**Keywords:** Tai Chi/Qigong intervention changes effects on cancer, cognition, physical activity, wellbeing, survivorship

## Abstract

**Introduction:**

Cognitive decline is a significant, persistent issue among breast cancer survivors (BCSs) affecting more than 50% and greatly impacting health and wellbeing, particularly among those who are aging. Scalable, lifestyle interventions to mitigate cognitive decline in this population are needed. This study aimed to explore the effects of Tai Chi Easy (TCE) on perceived cognition function among older BCSs.

**Methods:**

The current work was part of a larger NCI-funded trial (R01CA182901, primary results reported elsewhere). Female BCSs, aged 45–75 years, were randomized to one of three conditions: two active interventions, Tai Chi Easy (TCE) or sham Qigong (SQG), or an education control group (EDC). In this exploratory analysis of older female participants (aged 60–75y), perceived cognitive function and performance and related factors (including anxiety, depression, and insomnia symptom severity) were examined. We anticipated TCE and SQG would show greater improvements in perceived cognitive function and performance compared to EDC.

**Results:**

A total of 75 female BCS were included in the analysis (TCE = 23; SQG = 22; EDC = 30). Linear mixed model results showed BCS randomized to TCE and SQG reported improvements in perceived cognitive impairment and cognition-related effects on quality of life relative to EDC (with small to medium effect sizes; Cohen’s d = 0.46 and 0.76), whereas no results were found for cognitive performance measures. Relative to EDC, TCE did not exact changes in depression, anxiety, and insomnia symptom severity; however, SQG showed decreases in depression and insomnia symptom severity (with corresponding small to medium effect sizes, Cohen’s d = −0.36 and −0.56).

**Discussion:**

Findings from the current exploratory study suggest that low-exertion, gentle exercise, with or without breath and meditative focus, may improve perceived cognitive function, and, that without breath and meditative focus, may improve depressed mood and insomnia symptoms among older BCS. These promising findings may have immediate and long-term implications on accessible treatment options recommended by geriatricians and oncologists treating older BCS at an elevated risk for cognitive impairment.

**Clinical trial registration:**

The parent study was registered on ClinicalTrials.gov, identifier: NCT02690116.

## Introduction

There are more than 4 million breast cancer survivors (BCSs) in the United States (Ahles et al., 2022), and ~75% of this vulnerable population are survivors 60 years of age or older (Mandelblatt et al., 2018; Yang and Hendrix, 2018). Across the mass population of BCSs, >50% report cognitive decline post-treatment (i.e., chemotherapy, surgery); however, due to the paucity of clinical research among older BCSs current-day literature does not fully describe the prevalence and impact of cognitive impairment among older survivors.

Older BCSs are an exponentially growing population with unique and specific needs. Breast cancer represents the most frequently diagnosed cancer in the world (Wilkinson and Gathani, 2022) and the second leading cause of cancer death among women (Division of Cancer Prevention Control, 2023). Fortunately, due to notable advances in both diagnosis and treatment, long-term survival rates have increased, with current 5-year survival rates of ~90% and 10-year survival rates of approximately 80% (Whittaker et al., 2022). Despite these notable advancements, perceived cognitive impairment remains a frequently reported symptom during and beyond treatment (Lange et al., 2019). There is a steady and predictable inverse relationship between age and cognitive decline among older BCSs compared to younger BCSs (Root et al., 2023). Provided the increasing growth of the BCS population, and specifically the increasing number of older BCSs, understanding cognition-related issues specific to this older population is paramount.

Cognitive impairment is a significant and persistent issue among BCSs greatly impacting health, wellbeing, and quality of life, both immediately and long-term. Additionally, age-related cognitive decline, which includes declines in most skills, is one of the most well-known consequences and common experiences of aging (Salthouse, 2019). The cognitive effects of cancer treatment coupled with normal cognitive aging may increase the risk and further compound the effects of accelerated cognitive decline potentially impacting multiple domains of cognition and overall global cognitive health (Crouch et al., 2022; Root et al., 2023).

Cognitive decline can have a profoundly negative impact on quality of life, return to work/work productivity, relationships, childcare, daily responsibilities, self-worth, and ability to function independently. Cognitive impairments among BCSs are widespread, affecting most cognitive domains: memory, motor functioning, processing speed, attention, language (word-finding), and executive functioning (Lange et al., 2019; Dijkshoorn et al., 2021).

Common psychological and behavioral sequelae of breast cancer and breast cancer treatment among BCSs include psychological distress (i.e., anxiety, depression, fatigue) and insomnia (Bower, 2008). Psychological distress and insomnia are identified as potential modifiable risk factors for cognitive impairment (Boscher et al., 2020), and may, in part, contribute to pathophysiological mechanisms of cognitive impairment among BCSs (Yang and Hendrix, 2018). However, the contributing factors of cognitive impairment among BCSs are not fully understood and are considered to be complex and multifactorial, particularly for older BCSs. Older BCSs with higher levels of psychological distress are more likely to experience cognitive dysfunction (Crouch et al., 2022) making it critical to assess related factors in models exploring these relationships.

Meditative movement is a category of exercise including Tai Chi, Qigong and certain types of yoga connecting the mind, breath, and body to achieve deep states of relaxation (Larkey et al., 2009; Klein et al., 2016; Wayne et al., 2018; Osypiuk et al., 2020; James et al., 2022, 2023a; James et al., 2023b). Meditative movement practices have been shown to aid in symptom management in cancer patients and survivors by addressing both physical and psychological needs (Jahnke et al., 2010; Larkey et al., 2018; James et al., 2022; Rameshkumar et al., 2022). Meditative movement practices are often easier for BCSs to engage in compared to more traditional and intense aerobic (e.g., running, biking) and/or resistance training exercises (e.g., weightlifting) (Larkey et al., 2009; Soltero et al., 2023). Reportedly, BCSs respond well to low-exertion, gentle movements with a specific focus on awareness and integration of the breath (Larkey et al., 2015, 2016; Soltero et al., 2023). Previous research has demonstrated the benefits of Tai Chi and Qigong for improving commonly experienced post-cancer treatment symptoms and conditions among BCSs, including fatigue, cognitive impairment, disrupted sleep, depression, and increased body mass index (i.e., increased fat mass/decreased fat-free mass) (Larkey et al., 2016, 2018; Wayne et al., 2018; Low et al., 2020; Chang et al., 2023). Improving these noted outcomes can contribute to improved global health, wellbeing, and daily quality of life (Larkey et al., 2016; Chang et al., 2023).

BCSs are increasingly showing interest in complementary and alternative medicine modalities such as meditative movement practices (Stan et al., 2016); however, the body of research assessing the effects of meditative movement on cognitive functioning and impairment among older BCSs remains limited and often does not specifically test or examine effects in the oldest (age 60 and older) of survivors. At the same time, an evidence-based body of literature is accumulating suggesting that meditative movement interventions may slow cognitive decline or even improve early stages of Mild Cognitive Impairment among older adults (i.e., 60 years of age and older) (Zhang et al., 2018; Chan et al., 2019; Gu et al., 2021).

Tai Chi Easy (TCE) is a standardized and manualized type of meditative movement combining practices of Tai Chi and Qigong characterized by slow, low-exertion, flowing, rhythmic movements, breath work, and focused attention on the present moment, leading to a deep state of relaxation (Larkey et al., 2009; Smith et al., 2015; James et al., 2023a; James et al., 2023b). TCE involves a repeated series of movements aligning the body postures with the breath to achieve a relaxed state that can affect health outcomes in similar, as well as different, ways as traditional exercises (Jahnke et al., 2010). The goal of these integrated movements with breath focus is to cultivate one’s natural force or energy, also understood as Qi; this is considered the force that facilitates physiological and psychological mechanisms to enhance health, wellbeing and quality of life (Jahnke et al., 2010).

Currently, there are no evidence-based standards of care that promote neuroprotection and prevention of cognitive impairment among BCSs. While physical activity is recognized as a promising strategy to attenuate cognitive impairment (Hartman et al., 2018, 2021; Koevoets et al., 2022), the level of exercise intensity tested in that body of research is generally moderate to vigorous (i.e., higher levels of exertion than is typical for many meditative movement practices) (Larkey et al., 2009; Smith et al., 2015). The majority of research exploring cognition among BCSs has been conducted among a younger/midlife population, and not older populations (Mandelblatt et al., 2018). Here, we explore a sub-population of a larger trial to assess changes in cognitive function specifically among older BCS ages 60 to 75 years old using TCE as the primary intervention. There is a critical need for the development of non-pharmacological interventions for cognitive decline to combat the deleterious and impactful symptoms, both short- and long-term, that cancer and cancer treatment coupled with aging have on older BCS’ cognitive function without the potential side effects of pharmaceuticals.

The purpose of the current exploratory study (findings from a larger parent trial reported elsewhere)^1^ was to assess the short-term (i.e., post- 8-week intervention) effect of gentle movement interventions, including TCE (gentle movement with meditative breath focus) and sham Qigong (SQG; gentle movement only) as compared to an education-based inactive control intervention (EDC) among older BCS (i.e., 60–75 years old) on the primary outcomes of cognitive function (e.g., perceived cognitive impairment/cognitive abilities, as well as objective cognitive performance) and secondary outcomes related to cognitive function, including mood (i.e., anxiety and depression symptoms) and insomnia symptom severity. Given the lack of evidence-based research exploring cognitive impairment among older BCSs, here we purposely selected our participant group of BCSs ages 60–75 years old to better understand the needs and outcomes of this population and provide research insights to support additional studies. We hypothesized that from pre- to post-intervention, the two active movement groups (i.e., TCE and SQG) would show greater improvements in cognitive function and mood (i.e., anxiety, depression) and decreased insomnia symptom severity as compared to the EDC group.

## Materials and methods

### Study overview

The parent three-arm randomized controlled trial (RCT) was designed to explore the impact of TCE and a similarly low-exertion, gentle exercise, SQG, as compared to EDC on fatigue, sleep, and mood (i.e., anxiety, depression) among 167 fatigued, post-menopausal female BCSs aged 45–75 (see text footnote^1^). The parent study included primary and secondary measures at baseline, post eight-week intervention, and 24-weeks post-intervention. The parent study, a three-arm RCT from which this subset of data was drawn, was registered at http://www.clinicaltrials.gov Identifier: NCT02690116) and the protocol was previously published (Larkey et al., 2015).

For purposes of the current exploratory study, we assessed a sub-sample of older female BCSs, ages 60–75 years old (cut point chosen at the mean age of our full sample), on select outcomes of cognitive function, mood (i.e., anxiety, depression), and insomnia symptom severity from pre- to post-eight-week intervention including all three study arms (i.e., TCE, SQG, EDC).

### Participants

Study participants were recruited primarily through two local hospital systems using mailed letters to potentially interested participants and direct referrals from participating oncologists. Research team members regularly attended tumor board meetings and visited breast cancer support groups at local hospital and cancer organizations. Additionally, the team developed advertisements for local newspapers/news channels and social media (i.e., Facebook). Potential participants were screened for eligibility by trained study staff via phone call using the following inclusion criteria: (1) female patients diagnosed with breast cancer, (Stage 0-III); (2) between 6 months and 10 years past primary treatment (i.e., chemotherapy, radiation, surgery); (3) 45–75 years of age; (4) post-menopausal; (5) able to speak and read English or Spanish, and (6) experiencing fatigue (scoring ≤ 50 on “Vitality” scale of Medical Outcomes Survey Short Form, SF-36) (Brown et al., 2011). For this exploratory study, only participants aged 60–75 years were included in the analyses. Exclusion criteria included the following: (1) unable to stand for ten-minute segments (e.g., wheelchair or walker bound and too weak and/or unable to walk); (2) prior and/or ongoing substantial experience with mind-body practices that combined slow, rhythmic body and breath work (e.g., Tai Chi/Qigong or certain types of yoga); (3) fatigue-related factors (e.g., working night shift, restless leg syndrome); (4) co-morbidities of uncontrolled diabetes, endocrine, or auto-immune disorders; (5) major clinical depression; (6) current use of medications that may interfere with fatigue and/or biomarkers (e.g., antihistamine, cyclosporin, corticosteroid); and (7) consumption of more than two alcohol drinks per day. Selective serotonin reuptake inhibitors/antidepressants were allowed due to the high prevalence of use and lower likelihood of interference with potential response to intervention.

### Procedures

All study procedures and materials were reviewed and approved by the Institutional Review Board at the associated/funded university. Prior to the start of the study, all participants provided written informed consent (obtained in-person) to participate in this study. Cohorts of five to 20 participants were randomized to one of the three study arms. These intervention conditions allowed for (a) the separation of effects of education alone without any physical activity component in the intervention (in contract to the two active conditions), and (b) to distinguish unique effects of the TCE mind-body practice as compared to the SQG intervention that included gentle movement at a similar level of intensity to TCE, but without the meditative and breath foci. For purposes of the current subset of data and respective exploratory analysis, short-term study outcomes were assessed including baseline (T1) and post- 8-week intervention (T2). Data collection was performed by trained research staff using identical procedures at baseline (T1), and post-eight-week intervention (T2); all self-report questionnaire data were entered directly into REDCap by participants.

### Randomization and blinding

Consented participants for each cohort were randomized using minimization randomization methods to balance factors most likely to be related to fatigue (as the parent study was powered and designed primarily to test effects on fatigue): high vs. low BMI and high vs. low physical activity. To determine the low and high PA categories for stratification, we used the mid-point score from prior research with the same/similar population of BCS participants. The Women’s Health Initiative Brief Physical Activity Questionnaire (WHI-BPAQ) is a self-report instrument used in the Women’s Health Initiative study of breast cancer survivors and was shown to have high correlation with accelerometry (0.73) and comparable validity, sensitivity and measurement bias compared to the widely accepted Physical Activity Recall (PAR) (Johnson-Kozlow et al., 2007). This instrument was used to assess initial level of physical activity (PA) to determine high and low categories for stratification (based on prior data establishing a mean of 8.4 MET/hours/week for fatigued breast cancer survivors) (Hong et al., 2007).

Participants were told they were participating in a “Recovery and Rejuvenation” study where they would be assigned to a gentle movement intervention (TCE or SQG) or the education group (EDC), all designed to examine symptoms and recovery, but they were not told which was expected to be superior. Study staff involved in data collection, entry/cleaning, and analysis were blinded to the participants’ group assignment. Study staff involved in intervention delivery did not collect or interact with data. The participants were debriefed about their assigned groups (i.e., unblinded) after the completion of the final data collection.

### Study groups

#### Tai Chi Easy (TCE) intervention

##### TCE group

The eight-week TCE intervention is a standardized, manualized program that requires formal training and certification program for instructors provided by the Institute of Integral Qigong and Tai Chi. In the TCE intervention, participants were taught a series of low-impact/intensity and low-exertion exercises based on Qigong and selected Tai Chi movements that were simple to learn and repeated throughout each one-hour class (once weekly for eight weeks) (James et al., 2022). Group-based classes were taught by trained instructors once a week and lasted for one hour. Over the duration of the study, and across all 22 cohorts, all study classes were taught at community locations (e.g., community center, cancer center, local hospital). Classes were offered on different days/times (per cohort) and at different area locations to best accommodate the participant population. TCE participants were encouraged to spend 30 min/day “most days per week” engaging in at-home practice. To guide their at-home practice, participants received a professionally produced DVD and manual demonstrating a core set of ten TCE movements along with a hard copy tracking sheet to log their at-home practice time.

#### Sham Qigong group (SQG)

##### Active control group

The SQG was designed to provide a very similar type (gentle movement) and level of exercise, engagement, and energy expenditure compared to the TCE intervention group; however, this intervention deliberately omitted the focus on mind/body interaction, breath work, or meditative centering. A series of gentle movements similar to the actions and intensity of the qigong protocol, but without a focus on the breath or meditative state, were taught weekly (1 h) to participants by trained study staff. Participants were allowed to sit or stand for the duration of the class. The SQG protocol was developed by an exercise physiologist trained in cancer rehabilitation exercise. For purposes of at-home practice, a professionally produced DVD was provided which included the specific instructions for movement. Participants were encouraged to practice 30 min “most days of the week” at home.

#### Education control group (EDC)

##### Inactive control group

The EDC consisted of group-based activities (i.e., reading, group discussions) designed to provide brief education and group discussion for participants related to health, wellbeing, and quality of life as a BCS. The format of the ECG was conducted similar to a “book club” format with assigned weekly readings and discussion topics specific to breast cancer. Trained study staff facilitated weekly group sessions, which were based on the select reading materials specific to being a BCS. Each week a specific topic was used to guide the content questions and group discussion. Class times and at-home readings were designed to be equivalent in dose to the TCE class times and home practice. Thus, the EC group sessions lasted ~1 h, and were delivered at select community sites once per week. Similarly, participants were given additional readings to complete at home. Participants in the ECG were encouraged to complete weekly at-home readings to facilitate group discussions in classes.

### Measurements

#### Perceived cognitive function

Functional Assessment of Cancer Therapy-Cognitive Function version 3 (FACT-COG3) is a self-report measure of cognitive function, developed and validated in adult cancer patients with chemotherapy-induced cognitive problems. FACT-COG3 contains 33 items and asks participants to rate the frequency of each statement based on their experiences in the past week with the use of a five-point Likert scale. Higher scores indicate greater levels of perceived cognitive impairment. FACT-Cog consists of three subscales: Perceived Cognitive Impairments (PCI); Impact of Perceived Cognitive Impairment on Quality of Life (PCIQoL); and Perceived Cognitive Abilities (PCA) (Cronbach’s α = 0.96; 0.91; and 0.92, respectively) (Koch et al., 2023).

##### Cognitive performance

The Letter Number Sequencing (LNS), a subtest of the Wechsler Adult Intelligence Scale, Third Edition (WAIS-III), is a verbal/auditory task measure of attention and working memory. The task includes seven items with each item containing a string of three numbers and/or letters. This task requires participants to listen to a series of letters and digits, and then repeat back the stimuli with the digits in ascending numerical order followed by the letters in alphabetical order (Wechsler, 1997). The series of letters and digits increases in length as the task progresses. Digit Span, a subtest of the WAIS-III, is an additional verbal/auditory measure of attention and working memory. Digit Span has two subsections: Digits Forward and Digits Backward (DSF-DSB), each containing eight items. Participants are read a string of digits by the examiner and asked to repeat the digits either forwards or reverse. As the items progress, the task becomes more challenging as the span of digits increases (Wechsler, 1997). For LNS, DSF, and DSB, the tasks are discontinued after errors on all trials within an item.

##### Anxiety/depression

The Profile of Moods- Short Form (POMS-SF) is a 37-item self-report measure of psychological distress. The POMS-SF uses a five-point Likert scale (1 = not at all to 5 = extremely), such that lower scores indicate more favorable change in direction. The full POMS-SF comprises six subscales with Cronbach’s α ranging from 0.80 to 0.90 (Curran et al., 1995). Respective of decreasing participant burden and focusing on select moods related to the primary outcomes, only the Tension-Anxiety and Depression-Dejection subscales were included in this study. The Tension-Anxiety subscale captures the state of intensified physical and emotional tension caused by anxiety. The Depression-Dejection subscale evaluates components related to depression and self-inadequacy including sadness, loneliness, guilt, worthlessness, and hopelessness (Lin et al., 2014).

##### Insomnia symptom severity

The Insomnia Severity Index (ISI) is a self-report measure used to evaluate the presence and severity of insomnia symptoms within the past 2 weeks (Morin et al., 2011). The seven-item measure utilizes a five-point Likert scale (e.g., 0 = no symptom; 4 = severe symptom). Each item is summed for a total score ranging from 0 to 28 with higher scores indicating clinically significant insomnia symptoms. Scores ranging from 0–7 indicate the absence of clinically meaningful insomnia symptoms. Scores ranging from 8 to 14 indicate subthreshold or probable insomnia. Scores ranging from 15 to 21 indicate moderate clinical insomnia symptoms and scores ranging from 22 to 28 indicate severe clinical insomnia symptoms (Cronbach’s α = 0.84) (Bastien et al., 2001).

#### Statistical analysis

Participant characteristics at baseline were assessed for similar distribution across group allocation on key variables. To assess the short-term effect of TCE and SQG as compared to the EDC group among older BCS on the outcomes of cognitive function, mood (i.e., anxiety, depression), and insomnia symptom severity, linear mixed models (multilevel models) with time (level 1) nested within participants (level 2) was used for each outcome. Linear mixed models are appropriate to handle longitudinal data in which correlations between observations within each participant were expected. In all linear mixed models, random variation in the initial status was allowed (random-intercept model with the identity covariance structure). Time was treated as categorical in all models to compare baseline with 8 weeks. Additional computations examined effect size within-group changes from pre to post interventions. Between group effect sizes were determined as follows: small = 0.2 to 0.4; medium = 0.5 to 0.7; large = 0.8 to1.0 (Sullivan and Feinn, 2012). Stata version 17.0 was used for all analyses.

## Results

### Participants

**FIGURE 1:**
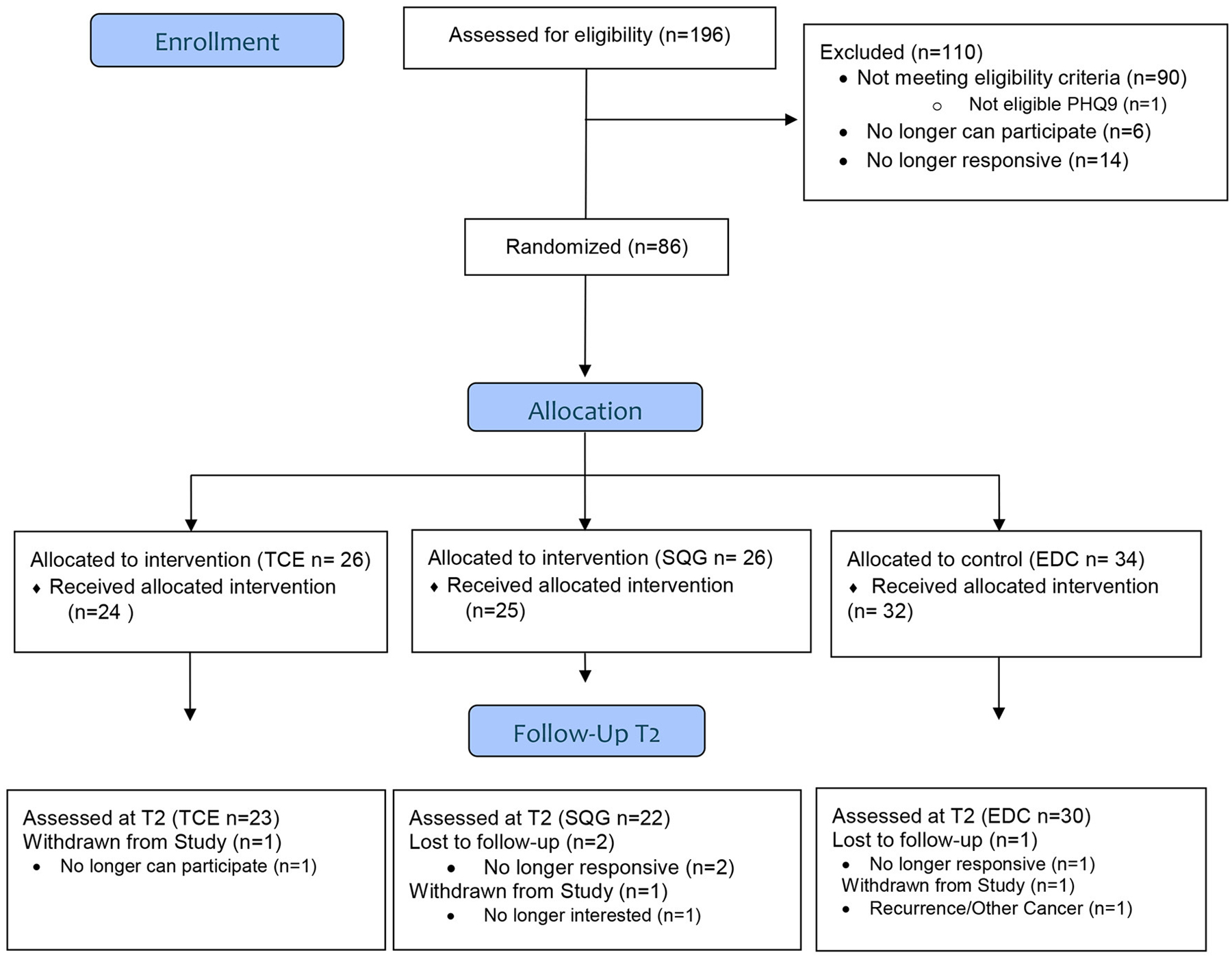
CONSORT flow diagram.

Of the parent study population (N = 167), 75 BCS, classified as older adults (mean ± SD; 67 ± 4 years) were included in this exploratory analysis. In this subsample, 23, 22, and 30 BCS were randomized to the TCE, SQG, and EDC arms, respectively (see Figure 1). Participant descriptives are reported in Table 1. The majority of participants had a graduate degree, were initially diagnosed with Stage 1 breast cancer, and were 3 years post-treatment at the time of eligibility screening. At baseline, characteristics of the sample (age, race/ethnicity, income, education, treatment history, cancer stage and mean physical activity levels) were fairly evenly distributed across treatment groups.

### Outcome results

#### Cognitive factors

Measures of cognitive function were assessed at baseline and 8-weeks for the TCE and SQG groups as compared to the EDC (Table 2). Results on perceived cognitive function all improved in the expected direction with variation in significance (displayed in Table 2, but not reported as significant due to the exploratory nature of this sub-study). Perceived Cognitive Ability showed only minimal improvement for TCE and SQG compared to EDC, additionally Perceived Cognitive Impairments and Perceived Cognitive Impairments on Quality of Life improved. The two WAIS-III assessments of cognitive performance, DSF/DSB and LNS showed no differences with minimal change when comparing TCE and SQG to EDC.

Given the preliminary and exploratory nature of these sub-group analyses (i.e., not powered for significance) effect sizes were computed to examine the magnitude and relevance of these changes. These effects sizes are computed for within-group changes and reported in Table 3. Small effect sizes were demonstrated for Perceived Cognitive Impairments and Perceived Cognitive Impairments on Quality of Life responses to TCE (Cohen’s d = 0.38 and.46 respectively), and small to medium effects for COG-PCA, COG-IMP, and COG QOL for SQG (Cohen’s d = 0.29, 0.52, 0.76, respectively). For WAIS-III performance tests, there was a small effect size (Cohen’s d = 0.45) for LNS in the SQG group. All remaining Cohen’s d for within-group self-reported cognitive function measures and WAIS-III performance test change were negligible (< 0.20).

#### Other factors related to cognitive function

In the linear mixed model analysis, TCE tests for change over time in depression, anxiety, and insomnia symptoms were not significant while SQG showed significant decreases relative to EDC in depression and insomnia symptom severity (with corresponding small to medium effect sizes, Cohen’s d = −0.36 and −0.56). Within arm changes for TCE showed a small effect size on anxiety (Cohen’s d = 0.38), and SQG and EDC showed small within-arm changes in depression (Cohen’s d = 0.35 and 0.36, respectively).

## Discussion

Due to notable advancements in oncology treatments, breast cancer survival rates are increasing; a respective priority of this field currently focuses on issues of survivorship and improvements in post-cancer quality of life. Interventions designed to help BCS mitigate cognitive impairment, while simultaneously improving quality of life are critical to support health and wellbeing throughout and beyond the years of cancer treatment. Gentle exercise interventions comprised of low-exertion movement may be highly adaptable and easily integrated protocols for improved cognition and wellbeing among older BCSs.

Our exploration of effects of two low-exertion activities, meditative movement (Tai Chi Easy) and a gentle movement intervention (SQG) compared to an inactive, education control condition (EDC) provided a nuanced look at the potential for movement to impact cognition in this selection of older BCS. While the linear mixed model showed improvements in perceived cognitive function and impact on quality of life (two of the three FACT-Cog subscales used in this study) for TCE as well as the SQG intervention, there appears to be a slight advantage conferred by the TCE (as implied by the somewhat higher coefficients for TCE over time as compared to EDC than SQG). In contrast, exploration of the effect sizes for change pre-to-post intervention per group in the cognitive factors, SQG fared better with effect sizes for perceived cognitive impairment and effects on quality of life (with TCE demonstrating only a small effect), and SQG showed an additional small effect for one of the performance measures, LNS.

While effects are still in the small range for most of the within-arm changes, it is interesting to note that the TCE showed a small effect on anxiety, while SQG effects appeared for depression and sleep factors. A number of studies and even meta-analyses, have indicated Traditional Chinese Medicine-based meditative movement practices (tai chi and qigong) fairly consistently improve depressive symptoms and sleep in cancer survivors (Larkey et al., 2016, 2018; Wayne et al., 2018; Low et al., 2020; Chang et al., 2023). In our current analysis, separating out the older BCS, there was minimal effect on depression and sleep, but rather showed effects on anxiety. While one of the key components of TCE is the slow, rhythmic movements with breath practice designed to clear and calm the mind, it is possible that these aspects of practice had more effect on the emotional state of tension or anxiety in these older survivors, while the movement without the additional breath and meditative practice was more successful for depressive mood and sleep. It is unclear why these would differ in effects.

Overall, the small to medium effects indicate potential for these gentle exercise practices to hold promise for BCSs who struggle with cognition and factors associated with cognitive function as they age. None of the cognition factors responded to the EDC condition suggesting that despite other advantages of educational support, this inactive control does little to support cognitive function.

### Limitations

The current exploratory analysis addresses a significant and oftentimes persistent issue among older BCSs specific to cognitive function, cognitive impairment and associated outcomes of mood and insomnia; however, there are noted limitations. As an exploratory analyses including a sub-group from the larger parent study, this work lacked power with only 23–30 participants per group. While we provided an analysis with the effect sizes within each group to sidestep sample size limitations, these findings are less meaningful for comparisons across groups. Also, the use of subjective self-report measures of cognitive function may be inherently vulnerable to over- or under-reporting of symptoms. Similarly, the subjective self-report measures of insomnia and mood are also vulnerable to perception bias. Overall, the outcomes are limited by the nature or being self-report, with the exception of the WAIS-III subtests and the study lacks comparisons of the cognitive function measures from a more and robust and varied panel of objective performance measures. Finally, the work was only conducted in the Southwest region of the United States, presenting a geographic limitation to the study findings which may yield results not generalizable across larger and more diverse samples.

### Future research directions

Two areas for future research directions are suggested: (1) cognitive training and (2) cognitive intra-individual variability (cognitive IIV). First, given the positive results found in our study on cognition, mood, and sleep, several other interventions may be combined with TCE, SGQ, and/or EDC to maximize such therapeutic effects. In an integrative review of 21 cognitive intervention studies in BCS, including seven cognitive training studies, researchers found that cognitive training was effective, at least in the short term, to improve the cognitive domains that were targeted for training (i.e., speed of processing training improved speed of processing, executive functioning training improve executive functioning) (Vance et al., 2017). For example, in a study of 60 BCSs, Meneses et al. (2018) randomized participants into either a 10-h speed of processing training group or into a no-contact control group. Researchers found that participants in the training group improved in this cognitive domain as well as in cancer-related symptoms (Bail et al., 2020). It is likely that the mechanisms in which cognitive training improved cognition and other effects are different than how TCE, SGQ, and/or EDC produce such therapeutic effects; so combined, they could synergize to maximize therapeutic outcomes. Second, recent research suggests that examining mean-based cognitive measures as conducted in our study and others may only capture some of the utility of such cognitive measures. In other words, the variability or spread/inconsistency in cognitive responses can be more predictive than summary or average cognitive scores. Prior research in aging and HIV suggests that such cognitive intra-individual variability is predictive of cortical atrophy, cognitive functioning 3 years later, progression to dementia, and even mortality (Vance et al., 2022). In a systematic review of four studies that used cognitive IIV, results suggest that it may be a way to explore subtle cognitive impairments (Vance et al., 2023). In fact, in a recent study of speed of processing training in adults with HIV, researcher explored whether a participant’s baseline cognitive IIV could modify training effects; they found that low vs high cognitive IIV could impact the timing in which treatment effects emerged (Vance et al., 2024). Our study and future studies should explore how such cognitive intra-individual variability may be used as an additional cognitive outcome as well as predictive variable for other non-cognitive outcomes (i.e., sleep, mood).

### Conclusion

Continued work is needed to understand the prevalence, severity, and impact of cognitive impairment among older BCSs. Findings from the current exploratory study suggest that exercise, even in the form of low-impact/exertion, gentle movement may be beneficial for improved cognitive function and associated outcomes (i.e., mood, insomnia) among older BCSs. These findings may have implications for geriatricians and oncologists working with and treating older BCSs. There is a significant need for non-pharmacological interventions to combat the deleterious short- and long-term impact that cancer, cancer treatment, and aging may have on older breast cancer survivors’ cognitive function and related psychological distress and sleep disturbances. Such interventions could reduce or even eliminate the use of prescription medications that may have adverse side effects. Longitudinal RCTs testing evidence-based interventions among older BCSs are critical to identifying feasible and appropriate protocols to attenuate cognitive impairment and improve cognitive function. Interdisciplinary work is imperative to identify and establish non-pharmacological modifiable lifestyle interventions targeting cognition and related health outcomes among older BCSs without additional side effects characteristic of many medications. Improvements across these respective outcomes may significantly increase immediate and long-term health, wellbeing, and quality of life for older BCSs at an elevated risk of cognitive impairment.

## Figures and Tables

**TABLE 1:** Participant descriptives.

	A (EDC) *n* = 30	B (TCE) *n* = 23	C (SQG) *n* = 22	Total *N* = 75
	Mean (SD) or Freq. (%)	Mean (SD) or Freq. (%)	Mean (SD) or Freq. (%)	Mean (SD) or Freq. (%)
Age (years)	67.1 (4.4)	67.3 (4.7)	67.0 (3.8)	67.1 (4.3)
**Education**				
<Graduate degree	18 (60.0%)	15 (65.2%)	11 (50.0%)	44 (58.7%)
≥Graduate degree	12 (40.0%)	8 (34.8%)	11 (50.0%)	31 (41.3%)
**Household Income (declined = 19)**				
<$25,000/year	1 (20%)	1 (20%)	3 (60%)	5 (6.6%)
$25,000–74,999/year	14 (48.3)%	11 (37.9%)	7 (24.2%)	29 (38.7%)
>$75,000 and up	8 (36.4%)	9 (40.9%)	5 (22.7%)	22 (29.4%)
**Ethnicity**				
Identify as Latina	4 (5.3%)	6 (8.0%)	4 (5.3%)	14 (18.7%)
**Race (declined = 5)**				
White	26 (39.3%)	21 (31.8%)	19 (28.8%)	66 (88%)
African American	1	1	0	2 (2.7%)
Native American/Alaska Native	2	0	0	2 (2.7%)
**Treatment(s) received**				
Surgery	22 (31.0%)	28 (39.4%)	21 (29.6%)	71 (94.6%)
Chemotherapy	15 (40.5%)	10 (27.0%)	12 (32.4%)	37 (49.3%)
Radiation	23	14	17	54 (72.0%)
Time since the most recent treatment (years)	3.0 (2.4)	3.0 (2.4)	2.6 (2.6)	2.9 (2.4)
Physical Activity at baseline (WHI-BPAQ)	20.41 (16.1)	19,5 (20.8)	21.1 (21.6)	20.3 (19.3)
**Cancer stage**				
Stage 0	4 (13.3%)	2 (8.7%)	2 (9.1%)	8 (10.7%)
Stage 1	10 (33.3%)	13 (56.5%)	7 (31.8%)	30 (40.0%)
Stage 2	11 (36.7%)	5 (21.7%)	7 (31.8%)	23 (30.7%)
Stage 3	5 (16.7%)	3 (13.0%)	6 (27.3%)	14 (18.7%)

EDC, education control group; TCE, Tai Chi Easy; SQG, sham Qigong.

**TABLE 2:** Multilevel model outcomes.

	Fixed effects												Variance components	
		T2 (ref. time 1)	Intervention group (ref. EDC)		Interaction	Age	Graduate degree (ref non-graduate)	Time since treatment	Cancer stage (ref: stage 1)			Constant	Level 1: within-person	Level 2: initial status
		TCE (*N* = 23)	SQG (*N* = 22)	Time2*TCE	Time2*SQG				Stage 0	Stage 2	Stage 3			
PCA Coef. [95% C.I.]	−0.10 [−1.84; 1.64]	−2.16 [−5.87; 1.54]	−1.42 [−5.14; 2.30]	1.09 [−1.55; 3.73]	1.83 [−0.85; 4.50]	0.18 [−0.16; 0.52]	1.10 [−1.81; 4.02]	0.12 [−0.48; 0.72]	−1.54 [−6.47; 3.39]	−3.53 [−6.99; −0.06]	−1.29 [−5.32; 2.74]	6.07 [−16.89; 29.04]	32.86 [21.88; 49.35]	11.82 [8.52; 16.38]
PCI [95% C.I.]	0.45 [−3.12; 4.02]	−6.15 [−14.84; 2.53]	1.23 [−7.49; 9.96]	6.12 [0.70; 11.53]	5.51 [0.03; 11.00]	0.12 [−0.69; 0.93]	0.63 [−6.32; 7.58]	0.45 [−0.98; 1.88]	−1.46 [−13.23; 10.30]	−8.91 [−17.18; −0.65]	−3.97 [−13.59; 5.65]	44.29 [−10.51; 99.09]	195.89 [133.07; 288.38]	49.73 [35.87; 68.94]
PCIQoL [95% C.I.]	−0.30 [−1.37; 0.77]	−2.28 [−4.27; −0.28]	−0.90 [−2.90; 1.11]	2.34 [0.71; 3.97]	2.07 [0.42; 3.72]	0.05 [−0.12; 0.23]	0.06 [−1.48; 1.59]	0.12 [−20.00; 0.43]	0.08 [−2.52; 2.67]	−1.97 [−3.79; −0.15]	−0.29 [−2.41; 1.84]	9.52 [−2.57; 21.62]	8.50 [5.47; 13.19]	4.49 [3.24; 6.23]
DSF-DSB [95% C.I.]	0.43 [−0.64; 1.51]	0.83 [−1.32; 2.97]	0.10 [−2.06; 2.25]	−0.96 [−2.59; 0.68]	−0.30 [−1.95; 1.36]	−0.22 [−0.42; −0.03]	0.92 [−0.75; 2.59]	0.18 [−0.17; 0.52]	−2.49 [−5.31; 0.34]	−0.62 [−2.61; 1.36]	0.69 [−1.62; 3.00]	31.34 [18.18; 44.51]	10.47 [6.88; 15.95]	4.53 [3.26; 6.27]
LNS [95% C.I.]	0.40 [−0.45; 1.25]	0.05 [−1.32; 1.42]	−0.58 [−1.96; 0.80]	0.03 [−1.26; 1.33]	0.65 [−0.67; 1.96]	−0.10 [−0.22; 0.01]	0.19 [−0.83; 1.21]	0.09 [−0.11; 0.30]	−1.69 [−3.42; 0.03]	1.04 [−0.17; 2.25]	0.37 [−1.04; 1.78]	16.61 [8.75; 24.66]	3.32 [2.00; 5.52]	2.85 [2.05; 3.95]
POMS-DEP [95% C.I.]	0.83 [−0.01; 1.68]	0.73 [−0.61; 2.08]	1.02 [−0.34; 2.37]	−1.27 [−2.55; 0.01]	−1.56 [−2.86; −0.26]	−0.07 [−0.18; 0.05]	0.30 [−0.70; 1.30]	−0.02 [−0.23; 0.18]	−0.71 [−2.40; 0.98]	0.44 [−0.74; 1.63]	0.61 [−0.77; 1.99]	13.28 [5.41; 21.15]	3.16 [1.89; 5.27]	2.78 [2.00; 3.85]
POMS-ANX [95% C.I.]	0.17 [−1.04; 1.37]	0.94 [−0.90; 2.79]	0.13 [−1.72; 1.99]	−1.47 [−3.30; 0.36]	−0.53 [−2.38; 1.32]	−0.17 [−0.33; −0.01]	0.18 [−1.17; 1.53]	0.14 [−0.14; 0.42]	−1.38 [−3.67; 0.90]	0.82 [−0.78; 2.43]	0.67 [−1.19; 2.54]	19.57 [8.91; 30.24]	5.50 [3.19; 9.47]	5.68 [4.10; 7.88]
ISI [95% C.I.]	0.10 [−1.14; 1.61]	1.63 [−1.36; 4.61]	4.12 [1.12; 7.13]	−1.97 [−4.26; 0.32]	−2.92 [−5.24; −0.60]	−0.15 [−0.42; 0.12]	−1.43 [−3.75; 0.89]	−0.31 [−0.79; 0.17]	−2.05 [−5.98; 1.89]	0.29 [−2.48; 3.05]	−2.11 [−5.32; 1.11]	20.36 [2.03; 38.69]	20.25 [13.27; 30.88]	8.88 [6.40; 12.31]

EDC, education control; TCE, Tai Chi Easy; SQG, sham Qigong; PCA, Perceived Cognitive Abilities; PCI, Perceived Cognitive Impairments; PCI-QoL, Perceived Cognitive Impairment on Quality of Life; DSF-DSB, Digit Span Forward, Digit Span Backward; LNS, Letter Number Sequence; POMS-DEP, Profile of Moods Scale—Depression; POMS-ANX, Profile of Moods Scale—Anxiety; ISI, Insomnia Severity Index.

**TABLE 3:** Effect size outcomes indicating within study arm (pre- and post-intervention changes).

	A (EDC) *N* = 30				B (TCE) *N* = 23				C (SCG) *N* = 22			
	T1	T2	Difference	Cohen’s d [95% C.I]	T1	T2	Difference	Cohen’s d [95% C.I]	T1	T2	Difference	Cohen’s d [95% C.I]
	Mean (SD)	Mean (SD)			Mean (SD)	Mean (SD)			Mean (SD)	Mean (SD)		
PCA	17.33 (6.75)	17.23 (7.32)	−0.10	−0.01 [−0.24; 0.21]	15.79 (6.61)	16.78 (7.27)	0.99	0.14 [−0.10; 0.38]	16.05 (5.47)	17.77 (6.32)	1.73	0.29 [−0.23;0.82]
PCI	50.03 (15.96)	50.48 (17.89)	0.45	0.03 [−0.20; 0.25]	45.39 (17.57)	51.96 (16.73)	6.57	0.38 [0.11; 0.65]	51.18 (12.62)	57.15 (10.10)	5.96	0.52 [.008;0.97]
PCIQoL	12.80 (3.70)	12.50 (3.61)	−0.30	−0.08 [−0.39; 0.22]	10.83 (4.87)	12.87 (4.06)	2.04	0.46 [0.11; 0.80]	11.91 (2.47)	13.68 (2.19)	1.77	0.76 [0.20;1.32]
DSF-DSB	16.97 (4.09)	17.40 (3.78)	0.43	0.11 [−0.25; 0.47]	17.87 (4.10)	17.35 (3.79)	−0.52	−0.13 [−0.41; 0.14]	17.32 (4.13)	17.45 (3.84)	0.14	0.03 [−0.24;0.31]
LNS	10.17 (3.00)	10.57 (2.87)	0.40	0.14 [−0.22; 0.49]	10.09 (2.45)	10.52 (2.17)	0.43	0.19 [−0.22; 0.60]	9.64 (2.04)	10.68 (2.53)	1.05	0.45 [0.09;0.82]
POMS-DEP	9.13 (1.61)	9.97 (2.92)	0.83	0.35 [0.04; 0.67]	9.78 (2.66)	9.35 (2.93)	−0.43	−0.16 [−0.53; 0.22]	10.27 (2.27)	9.55 (1.71)	−0.73	−0.36 [−0.88;0.16]
POMS-ANX	8.93 (3.67)	9.10 (3.78)	0.17	0.04 [−0.32; 0.41]	9.74 (3.72)	8.43 (3.12)	−1.30	−0.38 [−0.73; −0.03]	9.14 (3.52)	8.77 (1.90)	−0.36	−0.13 [−0.61; 0.35]
ISI	8.10 (5.42)	8.20 (5.19)	0.10	0.02 [−0.25; 0.29]	9.91 (6.24)	8.04 (5.87)	−1.87	−0.31 [−0.63; 0.01]	12.09 (5.14)	9.27 (4.84)	−2.82	−0.56 [−0.95;−0.18]

Cohen’s d: small (0.2), medium (0.5) and large (0.8); bootstrap estimation (number of replications = 1,000).

EDC, education control; TCE, Tai Chi Easy; SQG, sham Qigong; PCA, Perceived Cognitive Abilities; PCI, Perceived Cognitive Impairments; PCI-QoL, Perceived Cognitive Impairment on Quality of Life; DSF-DSB, Digit Span Forward, Digit Span Backward; LNS, Letter Number Sequence; POMS-DEP, Profile of Moods Scale—Depression; POMS-ANX, Profile of Moods Scale—Anxiety; ISI, Insomnia Severity Index.

## Data Availability

The original contributions presented in the study are included in the article/supplementary material, further inquiries can be directed to the corresponding author.

## Footnotes

1 Larkey, L. L., James, D. L., Han, S. Y., Weihs, K., Jahnke, R., Oh, D., et al. (under review). Randomized controlled trial testing Tai Chi easy/Qigong and Sham Qigong on breast cancer survivors’ fatigue and associated symptoms. *Complementary Therapies in Clinical Practice*.
